# Zertifizierung der Deutschen Gesellschaft für Neurorehabilitation e. V., DGNR: Leistung und Qualität der „Zentren für Beatmungsentwöhnung in der neurologisch-neurochirurgischen Frührehabilitation“

**DOI:** 10.1007/s00115-023-01540-1

**Published:** 2023-09-05

**Authors:** Thomas Platz, Tobias Schmidt-Wilcke, Martin Groß, Christian Friederich, Marcus Pohl

**Affiliations:** 1 AG Neurorehabilitation, Universitätsmedizin Greifswald, Fleischmannstraße 44, 17475 Greifswald, Deutschland; 2 Institut für Neurorehabilitation und Evidenzbasierung, An-Institut der Universität Greifswald, BDH-Klinik Greifswald, Greifswald, Deutschland; 3 Neurologisches Zentrum, Bezirksklinikum Mainkofen, Deggendorf, Deutschland; 4 Institut für Klinische Neurowissenschaften und Medizinische Psychologie, Heinrich-Heine-Universität Düsseldorf, Düsseldorf, Deutschland; 5 Evangelisches Krankenhaus Oldenburg, Oldenburg, Deutschland; 6 Oldenburger Forschungsnetzwerk Notfall- und Intensivmedizin, Carl von Ossietzky Universität Oldenburg, Oldenburg, Deutschland; 7 TÜV Rheinland Cert GmbH, Köln, Deutschland; 8 VAMED Klinik Schloss Pulsnitz, Pulsnitz, Deutschland

**Keywords:** Weaning, Frührehabilitation, Strukturqualität, Prozessqualität, Zertifizierung, Weaning, Early rehabilitation, Structural quality, Process quality, Certification

## Abstract

**Hintergrund:**

Die Zertifizierung von Zentren für Beatmungsentwöhnung in der neurologisch-neurochirurgischen Frührehabilitation (NNFR) durch die Deutsche Gesellschaft für Neurorehabilitation (DGNR) ist seit dem 01.10.2021 möglich.

**Ziel der Arbeit:**

Die Ergebnisse der Zertifizierung von Einrichtungen im ersten Jahr nach Aufnahme des Verfahrens werden vorgestellt.

**Material und Methoden:**

Im Rahmen der Zertifizierung werden 28 Kriterien geprüft, darunter eine Reihe mandatorisch zu erfüllender Charakteristika der Einrichtung. Die Kriterien gliedern sich in Strukturkriterien (i = 7), Diagnostikkriterien (i = 6), Personalkriterien (i = 3), Kriterien der internen Organisation (i = 7) und Qualitätsmanagementkriterien (i = 5).

**Ergebnisse:**

Insgesamt 13 Zentren wurden im ersten Jahr zertifiziert, die zusammen über 283 Betten zur Beatmungsentwöhnung („weaning“) in der NNFR verfügen und im Jahr vor der Zertifizierung 2278 Personen im Weaning betreuten, im Median pro Einrichtung 134 (Bereich [Min-Max] 44–414). Nur selten war das Weaning nicht erfolgreich, sodass vor Entlassung auf eine Heimbeatmung eingestellt werden musste (invasive Heimbeatmung Median pro Einrichtung 10 Personen, Bereich 2–25; nichtinvasive Heimbeatmung Median 0 Personen, Bereich 0–57). Festgestellt wurde ein hohes Maß an Prozess- und Strukturqualität in den zertifizierten Zentren: Über alle Prüfbereiche hinweg waren die Prüfkriterien zu allermeist erfüllt (Median Erfüllungsgrad 86 %) bzw. erfüllt mit von den Auditor*innen dokumentierten Verbesserungspotenzialhinweisen (Median 11 %).

**Schlussfolgerung:**

Erfolgreiches Weaning in der NNFR und ein hohes Maß an Prozess- und Strukturqualität lassen sich anhand der Zertifizierungsergebnisse der Zentren belegen, die diesen integrativen Ansatz bei der Beatmungsentwöhnung verfolgen.

## Abstract (en)

**Background:**

Certification of centers for weaning from a ventilator in neurological neurosurgical early rehabilitation (NNER) by the German Society for Neurorehabilitation (DGNR) is possible since 1 October 2021.

**Objective:**

The results of certification of facilities in the first year after starting the procedure are presented.

**Material and methods:**

As part of the certification process 28 criteria are assessed including a set of mandatory characteristics of the facility. The criteria are divided into structural criteria (i = 7), diagnostic criteria (i = 6), personnel criteria (i = 3), internal organization criteria (i = 7), and quality management criteria (i = 5).

**Results:**

A total of 13 centers were certified in the first year, with a combined total of 283 beds for weaning from a ventilator in the NNER and served 2278 persons to be weaned from a ventilator in the year before certification, with a median of 134 per facility (range 44–414). Only rarely was weaning unsuccessful, requiring conversion to home mechanical ventilation before discharge (invasive home mechanical ventilation median per facility 10 persons, range 2–25; non-invasive home mechanical ventilation median 0 persons, range 0–57). A high level of process and structural quality was documented for the certified centers: across all areas of assessment, the individual certification criteria were met in the vast majority of cases (median degree of complete fulfilment 86%) or met with improvement potentials documented by the auditors (median 11%).

**Conclusion:**

Successful weaning in NNER and a high level of process and structural quality can be demonstrated by the certification results of centers that follow this integrative approach to weaning from a ventilator in a NNER setting.

## Hintergrund und Fragestellung

Im europäischen Vergleich nimmt Deutschland mit seinen Intensivkapazitäten populationsbezogen eine Spitzenposition ein. Die Anzahl der Intensivbetten pro 100.000 Einwohner („accessibility index“, AI) war bei einem Vergleich 14 europäischer Staaten in Deutschland (AI = 35,3) am höchsten, in Dänemark (AI = 6,4) am niedrigsten. Mit dem Zugang zu Intensivbetten negativ korreliert ist beispielsweise die fallbezogene Mortalität an COVID-19-Erkrankter (r = −0,57; *p* < 0,001; [1]). Eine leistungsfähige Intensivmedizin sichert also das Überleben kritisch Erkrankter. Nicht wenige beatmungspflichtige intensivmedizinisch Versorgte bedürfen einer prolongierten Beatmungsentwöhnungsbehandlung („weaning“). Innerhalb der Fachbereiche Pneumologie, Anästhesiologie und Neurologie sind jeweils spezialisierte Versorgungsstrukturen entstanden, die prolongierte Beatmungsentwöhnung durchführen. Nicht selten liegen dem prolongierten Verlauf des Weanings neurologische Erkrankungen zugrunde. Ursächlich hierfür sind unter anderem demographisch bedingte Veränderungen der Altersstruktur von Intensivpatienten [2]. Mit höherem Lebensalter steigt das Risiko, an einer neurologischen Erkrankung oder an einer neurologischen Komplikation einer – meist internistischen oder chirurgischen – Primärerkrankung (häufig „post intensive care syndrome“ [PICS] mit Critical-illness-Polyneuropathie/-Myopathie) zu erkranken. Häufig sind dann eine Beatmung auf einer Intensivstation sowie eine anschließende neurologisch-neurochirurgische Frührehabilitation (NNFR) mit Beatmungs- und Trachealkanülenentwöhnung notwendig. So wurden bereits im Jahr 2009 in 7 NNFR-Kliniken insgesamt 1486 Patient*innen zum Weaning aufgenommen, 97,5 % der Aufnahmediagnosen waren neurologisch [3]. In der NNFR wird die Beatmungsentwöhnung als integraler Teil der Neurorehabilitation verstanden. Parallel zur Beatmungs- und Trachealkanülenentwöhnung werden durch den multiprofessionellen Ansatz der NNFR die oft komplexen Erkrankungsfolgen – z. B. in den Bereichen Bewusstsein, Kognition, Kommunikation, Sensorik, Motorik – spezifisch behandelt und die Alltagskompetenzen gefördert, die Pflegebedürftigkeit wird reduziert. Auf die klinischen Besonderheiten dieser Versorgung weist die S2k-Leitlinien zum prolongierten Weaning in der NNFR hin [4]. Dass mit kombinierter intensiv- und beatmungsmedizinischer sowie neurologisch frührehabilitativer Versorgung ein hoher Behandlungserfolg sowohl in Bezug auf Beatmungs- und Trachealkanülenentwöhnung als auch die Alltagskompetenz erreicht wird, wurde mehrfach im Rahmen multizentrischer Studien gezeigt [3, 5, 6]. Weaning in der NNFR ist somit sehr erfolgreich, unterstützt die Lebensqualität und Teilhabe der Versorgten maßgeblich und trägt wesentlich zur Vermeidung eines außerklinischen Intensivpflegebedarfs bei. Mit ca. 1100 Weaning-Betten in der NNFR in Deutschland handelt es sich um ein zentrales Versorgungssegment [7].

Wie Spezialisierung und qualitativ hochwertige Versorgung in diesem Bereich objektiv nachgewiesen werden kann, zeigt die kürzlich etablierte Zertifizierung für „Zentren für Beatmungsentwöhnung in der neurologisch-neurochirurgischen Frührehabilitation“ durch die Deutsche Gesellschaft für Neurorehabilitation (DGNR) [8]. Zertifizierungen ermöglichen durch Vereinheitlichung von Standards eine konzeptionelle und qualitative Transparenz. Sie vereinfachen dadurch die Diskussion über erforderliche Strukturmerkmale, Behandlungsinhalte, Kapazitäten und Finanzierung.

Die Zertifizierung von Zentren für Beatmungsentwöhnung in der NNFR durch die DGNR ist seit dem 01.10.2021 möglich. Die Zertifizierungskriterien beschreiben ein Anforderungsprofil, das für eine fachgerechte und qualitätsgesicherte Versorgung von Beatmungspatienten in der NNFR steht. Das Zertifikat berücksichtigt die strukturellen Unterschiede der in der NNFR tätigen Einrichtungen und kann sowohl durch Facheinrichtungen (Fachkrankenhäusern) als auch durch Frührehabilitationsabteilungen an Akutkrankenhäusern (Krankenhäuser der Regel‑, Schwerpunkt- oder Maximalversorgung) erworben werden.

In diesem Beitrag werden Leistungen, Struktur- und Prozessmerkmale der ersten (13) innerhalb eines Jahres durch die DGNR zertifizierten Zentren für Beatmungsentwöhnung in der NNFR mitgeteilt.

## Studiendesign und Untersuchungsmethoden

### Zertifizierungsablauf

Die Durchführung der Zertifizierung erfolgt analog zur Zertifizierung von Stroke-Units der Deutschen Schlaganfall-Gesellschaft (https://www.dsg-info.de/stroke-units-neurovaskulaere-netzwerke/) in Zusammenarbeit mit dem TÜV Rheinland. Zunächst sendet die Einrichtung den Erhebungsbogen an den TÜV Rheinland (Erhebungsbogen erhältlich unter https://www.dgnr.de/zertifizierung/zertifizierung-info). Anschließend erfolgt die Begehung durch eine/einen vom TÜV Rheinland gestellte/n leitende/n Auditor*in und eine/n Fachauditor*in der DGNR. Deren Bericht wird mit entsprechender Empfehlung dem Zertifizierungsausschuss der DGNR zur finalen Begutachtung (zur Erteilung bzw. Nichterteilung des Zertifikats) vorgelegt.

### Zertifizierungskriterien

Im Rahmen der Zertifizierung werden 28 Kriterien geprüft, darunter eine Reihe mandatorisch zu erfüllender Charakteristika der Einrichtung. Die Vergabe des Zertifikats setzt voraus, dass sowohl die medizinischen Standards der Beatmungsentwöhnung in der NNFR als auch wesentliche in der ISO 9001:2015 festgelegten Vorgaben des betrieblichen Qualitätsmanagements [9] erfüllt werden. Die Kriterien gliedern sich in Strukturkriterien (i = 7), Diagnostikkriterien (i = 6), Personalkriterien (i = 3), Kriterien der internen Organisation (i = 7) und Qualitätsmanagementkriterien (i = 5). Die Kriterien im Einzelnen sind in Tab. 1 aufgeführt.

**Table 1:** 

*Strukturkriterien (i* *=* *7)*	Allgemeine Informationen zur Einrichtung
	Anzahl an Betten
	„Palliative care“
	Leistungsdaten des Zentrums für Beatmungsentwöhnung im Bezugsjahr (letztes Kalenderjahr)
	Diagnosen der Beatmungsentwöhnungspatienten/Fallzahl
	Organersatz‑/Unterstützungsverfahren/Fallzahl
	Ausstattung (Bettplätze und Station)
*Diagnostikkriterien (i* *=* *6)*	Routinelabor
	Endoskopische Verfahren
	Neurologische Funktionsdiagnostik
	Ultraschallgerät, mit diversen Funktionen
	Radiologische Bildgebung
	Respiratorische Diagnostik
*Personalkriterien (i* *=* *3)*	Ärztliche Leitung der NNFR
	Ärztliche Leitung der Beatmungsentwöhnungseinheit
	Kommunikation, Einarbeitung und Fortbildung
*Interne Organisationskriterien (i* *=* *7)*	Medizingeräte nach Medical Device Regulation
	Medikamentenmanagement
	Hygienemanagement
	Regelmäßige Pflichtschulungen
	Schriftliche Konzepte für spezifische Prozeduren
	Spezielle Qualifikationen des im Zentrum für Beatmungsentwöhnung tätigen Personals
	Mitbehandlung in der Einrichtung gewährleistet für die wesentlichen Disziplinen
*Qualitätsmanagementkriterien (i* *=* *5)*	Sind die Abteilung/Klinikum oder einzelne Bereiche bereits nach anderem QM-System zertifiziert?
	Ist in der Einrichtung ein Konzept zum klinischen Risikomanagement vorhanden?
	Führt die Abteilung/Klinik im Bereich der Beatmungsentwöhnungseinheit regelmäßig Selbstbewertungen/interne Audits durch?
	Führt die Abteilung/Klinik im Bereich der Beatmungsentwöhnungseinheit regelmäßig Selbstbewertungen der Todesfälle durch (sog. internes Todesfallreview)?
	Gibt es ein strukturiertes Aufnahme- und Verlegungsmanagement?

*NNFR* neurologisch-neurochirurgische Frührehabilitation, *QM* Qualitätsmanagement

Bei allen Kriterien wird jeweils von den Auditor*innen geprüft, ob Konformität besteht, ob die Einrichtungscharakteristika mit den Prüfkriterien in Übereinstimmung sind, also das jeweilige Prüfkriterium somit „erfüllt“ ist. Es kann vorkommen, dass ein Kriterium zwar erfüllt ist, aber dabei dennoch auch Verbesserungspotenzial erkannt und benannt wird; dies wird als „Verbesserungspotenzial“ kodiert. Bei Nichtkonformität werden die Kategorien „untergeordnete Nichtkonformität“ (zwar Kriterium nicht erfüllt, aber keine wesentliche Abweichung dadurch bedingt) bzw. „wesentliche Nichtkonformität“ (Nichtkonformität, die nicht mit den Zertifizierungsstandards vereinbar ist) unterschieden.

### Statistik

Die Ergebnisse der Zertifizierung im ersten Jahr nach Einführung derselben werden mittels deskriptiver Statistik dokumentiert.

Je nach Eigenschaft der erhobenen Kriterien wird die Verteilung der über die zertifizierten Einrichtungen erhobenen Merkmalsausprägungen quantitativ als absolute bzw. relative Häufigkeiten, Median, Interquartilenbereich („interquartile range“, IQR) und Minimum und Maximum des beobachteten Wertebereichs dokumentiert.

## Ergebnisse

**Figure 1:**
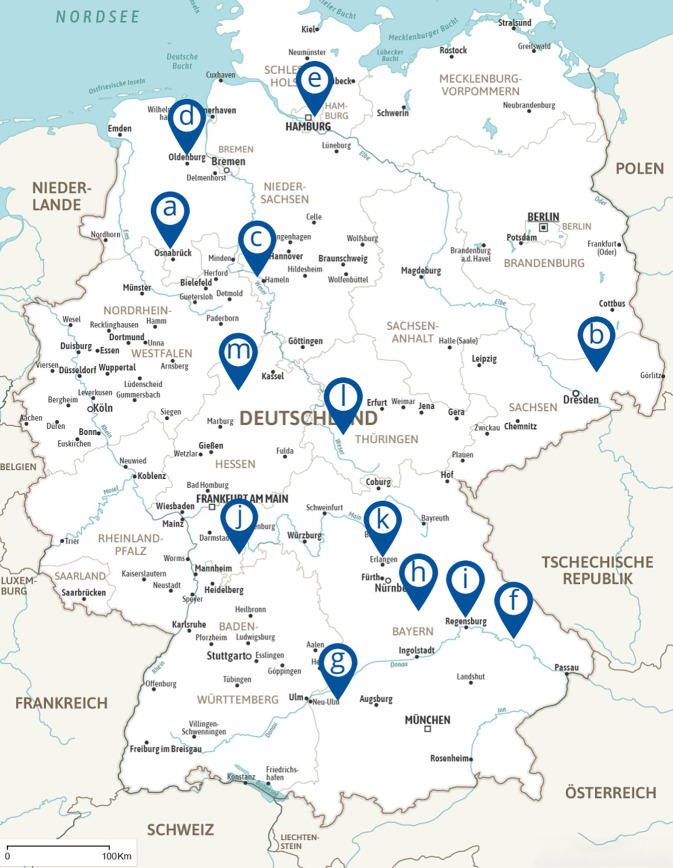

Im ersten Jahr nach Einführung der DGNR-Zertifizierung für „Zentren für Beatmungsentwöhnung in der neurologisch-neurochirurgischen Frührehabilitation“ haben sich 13 NNFR-Einrichtungen um ein Zertifikat bemüht und dieses im Rahmen des Zertifizierungsverfahrens erhalten. Ihre geographische Verteilung ist in Abb. 1 wiedergegeben.

Struktur- und Leistungsdaten der Zentren sind in Tab. 2 im Überblick aufgeführt.

**Table 2:** 

**Häufigkeiten *n* (%)**						
*Krankenhaustyp*	UNI (III*U*)	MAX (III)	R/SPV (II)	FK	GV	
	0 (0 %)	1 (8 %)	3 (23 %)	9 (69 %)	0 (0 %)	
*Palliativversorgung*	Station	Dienst	Team			
	2 (15 %)	3 (23 %)	12 (92 %)			
*Leitung NNFR – FA*	Neurologie					
	13 (100 %)					
*Leitung Weaning – FA*	Neurologie	Anästhesiologie	Innere Medizin			
	11 (84 %)	1 (8 %)	1 (8 %)			
*Leitung Weaning – ZB*	INT	N‑INT	NOTF	PALL	REHA	SOZ
	8 (62 %)	4 (31 %)	2 (15 %)	1 (8 %)	2 (15 %)	1 (8 %)
**Verteilungen**	**Median**	**IQR**	**Min–Max**	***n***		
*Betten – Beatmung-NNFR*	17	10–26	5–68	13		
*Betten – NNFR/NR o.B.*	63	41–79	12–171	13		
*Weaning-Fälle*	134	96–255	44–414	13		
*Beatm.-Std./Fall (Median)*	386	307–455	187–1027	13		
*Dialysefälle*	0	0–54,5	0–251	12		
*LVAD-Fälle*	0	0–0,5	0–7	12		
*HB-IVB-Fälle*	10	4–17,5	2–25	12		
*HB-NIV-Fälle*	0	0–3,5	0–57	12		
*Mortalitätsrate*	0,15	0,14–0,22	0,04–0,35	5		

Fallangaben beziehen sich auf das Kalenderjahr, das dem Zertifizierungsaudit vorausging. *n* bezieht sich auf die Anzahl der Einrichtung mit Darlegung der Daten im Auditbericht. Mortalitätsrate: Anzahl verstorbene/Anzahl entlassene Fälle

*Beatmung-NNFR* prolongierte Beatmungsentwöhnung in der neurologisch-neurochirurgischen Frührehabilitation, *FA* fachärztliche Qualifikation, *FK* Fachkrankenhaus, *GV (I)* Krankenhaus der Grundversorgung (Level I), *HB-IVB* Einstellung auf invasive Heimbeatmung, *HB-NIV* Einstellung auf nich-invasive Heimbeatmung, *INT* Intensivmedizin, *IQR* „interquartile range“, *LVAD* „left ventricular assist device“, *Min* Minimum, *Max* Maximum, *MAX (III)* Krankenhaus der Maximalversorgung (Level III), *N‑INT* neurologische Intensivmedizin, *NNFR/NR o.B.* neurologisch-neurochirurgische Frührehabilitation/Neurorehabilitation, ohne Beatmungsentwöhnung, *NOTF* Notfallmedizin, *PALL* Palliativmedizin, *UNI (IIIU)* Universitätsklinikum (Level III*U*), *REHA* Rehabilitationswesen, *SOZ* Sozialmedizin, *R/SPV (II)* Krankenhaus der Regel- bzw. Schwerpunktversorgung (Level II), *ZB* Zusatzbezeichnung

Überwiegend handelt es sich um Fachkrankenhäuser (69 %) und Krankenhäuser der Regel- und Schwerpunktversorgung (23 %).

Alle Einrichtungen wurden ärztlich von einer/m Fachärzt*in für Neurologie geleitet. Die Bereiche für Beatmungsentwöhnung wurden in 84 % ebenfalls durch eine/n Fachärzt*in für Neurologie geleitet, in 16 % lag ein kooperatives Leitungsmodell (zusätzlich Fachärzt*in für Anästhesiologie oder Innere Medizin) vor. Über eine intensivmedizinische Zusatzbezeichnung verfügten 93 % der ärztlichen Leitungen der Bereiche für Beatmungsentwöhnung.

Die zertifizierten Zentren verfügen über insgesamt 283 Betten zur Beatmungsentwöhnung in der NNFR und betreuten im Jahr vor der Zertifizierung 2278 Personen im Weaning. Die Bettenstärke für das Weaning in der NNFR variierte unter den zertifizierten Einrichtungen zwischen 5 und 68 Betten (Median 17), immer ergänzt um mindestens ebenso viele Betten der NNFR für nicht beatmete Patient*innen (Median 63, Bereich 12–171). Im Median wurden im Jahr vor der Zertifizierung pro Einrichtung 134 Personen im Weaning behandelt (Bereich 44–414). Nur selten war das Weaning nicht erfolgreich, sodass vor Entlassung auf eine Heimbeatmung eingestellt werden musste (Einstellung auf invasive Heimbeatmung: Median pro Einrichtung 10 Personen, Bereich 2–25; nichtinvasive Heimbeatmung: Median 0 Personen, Bereich 0–57).

Das Diagnosespektrum der zur Beatmungsentwöhnung in der NNFR aufgenommenen Patient*innen ist in Tab. 3 wiedergegeben.

**Table 3:** 

Verteilungen	Median	IQR	Min–Max
*PICS*	41,5	20–111,5	2–221
*Insult*	24,5	11,5–61	6–99
*IZB*	15	9,5–40,5	6–80
*HYPOX*	15	6,5–31,5	1–53
*SHT*	10,5	6–18	3–71
*SAB*	8,5	3,5–17	0–32
*Enzephalitis*	2,5	2–3	1–3
*GBS*	2	1–3,5	0–7
*ALS*	1	0–2	0–17
*Meningitis*	0,5	0–2,5	0–15
*Myasthenie*	0	0–1	0–4
*Sonstige*	14	5,5–18	0–38

Fallangaben beziehen sich auf das Kalenderjahr, das dem Zertifizierungsaudit vorausging. Angaben zu den Diagnoseverteilungen wurden von 12 der 13 zertifizierten Zentren mitgeteilt

*ALS* amyotrophe Lateralsklerose, *GBS* Guillain-Barré-Syndrom, *HYPOX* hypoxämische Hirnschädigung, *Insult* zerebral-ischämischer Insult, *IZB* nichttraumatische intrazerebrale Blutung, *PICS* „post intensive care syndrome“, *SAB* nichttraumatische Subarachnoidalblutung, *SHT* Schädel-Hirn-Trauma

Hier wird ein breites Spektrum neurologischer Erkrankungen deutlich, bei denen eine prolongierte Beatmungsentwöhnung im Setting einer spezialisierten NNFR-Einrichtung erforderlich war, u. a. auch häufig das „post intensive care syndrome“ (PICS) mit Critical-illness-Polyneuropathie/-Myopathie als neurologische Folgeerkrankung einer nichtneurologischen – meist internistischen oder chirurgischen – Primärerkrankung mit kritischem Krankheitsverlauf.

Über die verschiedenen Bereiche der Zertifizierungskriterien jeweils aggregiert ist der Erfüllungsgrade der Zertifizierungskriterien in Tab. 4 dargestellt.

**Table 4:** 

Verteilungen (in %)	Median	IQR	Min–Max
*Strukturkriterien (i* *=* *7)*			
Erfüllt	100	86–100	71–100
Verbess.potenzial	0	0–14	0–29
Nichtkonformität	0	0–0	0–0
Wesentl. NK	0	0–0	0–0
*Dignostikkriterien (i* *=* *6)*			
Erfüllt	100	100–100	83–100
Verbess.potenzial	0	0–0	0–17
Nichtkonformität	0	0–0	0–0
Wesentl. NK	0	0–0	0–0
*Personalkriterien (i* *=* *3)*			
Erfüllt	100	100–100	67–100
Verbess.potenzial	0	0–0	0–33
Nichtkonformität	0	0–0	0–0
Wesentl. NK	0	0–0	0–0
*Interne Organisationskriterien (i* *=* *7)*			
Erfüllt	71	57–71	29–100
Verbess.potenzial	29	14–43	0–71
Nichtkonformität	0	0–0	0–14
Wesentl. NK	0	0–0	0–0
*Qualitätsmanagementkriterien (i* *=* *5)*			
Erfüllt	80	60–80	40–100
Verbess.potenzial	20	20–40	0–60
Nichtkonformität	0	0–0	0–20
Wesentl. NK	0	0–0	0–0
*Alle Kriterien (i* *=* *28)*			
Erfüllt	86	82–89	68–96
Verbess.potenzial	11	11–18	4–32
Nichtkonformität	0	0–4	0–7
Wesentl. NK	0	0–0	0–0

*i* Anzahl der Kriterien, *Nichtkonformität* untergeordnete Nichtkonformität, *Verbess.potenzial* Kriterium zwar erfüllt, aber Verbesserungspotenzial erkannt, *Wesentl. NK* wesentliche Nichtkonformität

Über alle Prüfbereiche hinweg waren die (28) Prüfkriterien zu allermeist erfüllt (Erfüllungsgrad in den Einrichtungen: Median 86 %, Bereich [Min-Max] 68–96 %) bzw. erfüllt mit von den Auditor*innen dokumentierten Verbesserungspotenzialhinweisen (Median 11 %, Bereich 4–32 %). „Untergeordnete Nichtkonformitäten“, bei denen das Kriterium nicht erfüllt, aber keine wesentliche Abweichung dadurch bedingt ist, kamen nur selten vor (Median 0 %, Bereich 0–7 %). „Wesentliche Nichtkonformitäten“ wurden bei den Zertifizierungsverfahren in Gänze nicht beobachtet.

## Diskussion

Ein Jahr nach Einführung der DGNR-Zertifizierung für „Zentren für Beatmungsentwöhnung in der neurologisch-neurochirurgischen Frührehabilitation“ konnten bereits 13 hochspezialisierte Zentren zertifiziert werden. Zusammen verfügen sie über 283 Betten zur Beatmungsentwöhnung in der NNFR und betreuten im Jahr vor der Zertifizierung 2278 Personen im Weaning. Gemäß einer früheren Umfrage führen in Deutschland allerdings 69 Einrichtungen auf 1094 Betten die Beatmungsentwöhnung in der NNFR durch [7]. Somit sind die meisten Einrichtungen noch nicht zertifiziert, und die Anzahl der jährlich in Deutschland durchgeführten Beatmungsentwöhnungsbehandlungen in der NNFR liegt wahrscheinlich drei- bis vierfach höher als die oben genannten 2278 Fälle.

Das Diagnosespektrum der zur Beatmungsentwöhnung in der NNFR aufgenommenen Personen umfasst neben dem „post intensive care syndrome“ (PICS) mit Critical-illness-Polyneuropathie/-Myopathie verschiedene neurologische Erkrankungen, die mit schweren Körperfunktionsstörungen einhergehen können, darunter häufiger zerebral-ischämische Infarkte ebenso wie nichttraumatische intrazerebrale Blutungen und Subarachnoidalblutungen, hypoxämische Hirnschädigungen und Schädel-Hirn-Traumata. Das Spektrum entspricht dem erwarteten in der NNFR mit Weaning [3, 5, 6]. Der relativ hohe Anteil an Personen mit PICS und Bedarf für ein prolongiertes Weaning zeigt einmal mehr, dass die sehr leistungsfähige und gut ausgebaute Intensivmedizin in Deutschland das Überleben auch bei schwerem (Multi‑)Organversagen sichert, woraus allerdings ein hoher kombinierte Weaning- und Frührehabilitationsbedarf resultiert. Da sich diese Personengruppe durch ein hohes Maß an Multimorbidität auszeichnet, ist die Gewährleistung der medizinischen Versorgungsqualität, wie im Zertifizierungsverfahren hinterlegt, von besonderer Bedeutung. Dies trifft umso mehr zu, als bereits im Rahmen des demographischen Wandels absehbar ist, dass dieser Versorgungsbedarf in den nächsten Jahren weiter steigen wird [10, 11].

Die deskriptive Statistik des Erfüllungsgrades der Zertifizierungskriterien (vgl. Kriterien in Tab. 1) in Tab. 4 zeigt ein hohes Maß an Struktur- und Prozessqualität in den zertifizierten Zentren für Beatmungsentwöhnung in der NNFR. Die unabhängig dokumentierten Zertifizierungsdaten zeigten diesbezüglich auch wenig Variabilität über die zertifizierten Zentren für Beatmungsentwöhnung in der NNFR hinweg. Dies kann als wichtige Beobachtung erachtet werden, da die beteiligten Zentren mit durchaus unterschiedlichen Rahmenbedingungen arbeiteten. Beispielsweise variierten die Größe der Einrichtungen und damit die Anzahl der Behandlungsfälle im prolongierten Weaning nicht unerheblich. Zumindest, wenn das Kriterium der Mindestfallzahl von 40 begonnenen Beatmungsentwöhnungen pro Jahr erfüllt ist, wird auch bei unterschiedlichen großen Organisationseinheiten der Zentren für Beatmungsentwöhnung in der NNFR ein hohes Maß an Struktur- und Prozessqualität erreicht.

Der integrative Behandlungsansatz der NNFR zeichnet sich dabei vor allem dadurch aus, dass schon während der Beatmungsentwöhnung zum frühestmöglichen Zeitpunkt eine umfangreiche Förderung der Patient*innen durch rehabilitative Maßnahmen erfolgt. Erfolgreiches Weaning in der NNFR lässt sich anhand der Ergebnisse der Zentren belegen, die diesen integrativen Ansatz bei der Beatmungsentwöhnung verfolgen. Durch die NNFR wird die Unabhängigkeit, Selbstbestimmtheit, Teilhabe und Lebensqualität der Versorgten maßgeblich gefördert – damit trägt der Behandlungsansatz deutschlandweit auch wesentlich zur Vermeidung eines außerklinischen Intensivpflegebedarfs bei.

Eine Erweiterung des Leistungsspektrums der NNFR wird sich zukünftig durch die vermehrte Wiederaufnahme von Patient*innen aus der außerklinischen Intensivpflege zur Beatmungs- und Trachealkanülenentwöhnung in NNFR-Einrichtungen vollziehen. Bei neurologisch Erkrankten kann nämlich über einen längeren, z. B. mehrmonatigen Verlauf eine weitere funktionelle Erholung erreicht werden [12] mit resultierend verbesserten Voraussetzungen für einen nochmaligen Ansatz einer prolongierten Beatmungsentwöhnung oder einer Trachealkanülenentwöhnung in einer spezialisierten NNFR-Einrichtung. Hierdurch wiederum kann eine Verbesserung der Lebensqualität Betroffener erzielt werden sowie auch – bei Abwendung eines weiteren außerklinischen Intensivpflegebedarfs – eine erhebliche Kostensenkung für das Gesundheitssystem.

Perspektivisch werden sich, ähnlich wie bei der Zertifizierung der Stroke-Units, die Kriterien der DGNR-Zertifizierung für „Zentren für Beatmungsentwöhnung in der neurologisch-neurochirurgischen Frührehabilitation“ iterativ an neue Versorgungsstandards, aber auch an neue demographische und technologische Entwicklungen anpassen, um einen möglichst hohe Qualität der zertifikatstragenden Einrichtungen zu garantieren.

## Fazit für die Praxis

- Zentren für Beatmungsentwöhnung in der NNFR weisen ein hohes Maß an Struktur- und Prozessqualität auf.
- Schon während der Beatmungsentwöhnung erfolgt eine umfangreiche Förderung der Patient*innen durch rehabilitative Maßnahmen.
- Der integrative Ansatz bei der Beatmungsentwöhnung ist erfolgreich.
- Damit trägt der Behandlungsansatz deutschlandweit wesentlich zur Vermeidung eines außerklinischen Intensivpflegebedarfs bei.
